# Practical Tips for Diagnosis and Management of Concomitant Inflammatory Spondyloarthritis and Inflammatory Bowel Disease

**DOI:** 10.1007/s11894-025-00990-8

**Published:** 2025-07-14

**Authors:** Katherine Falloon, Michael Forney, Shashank Cheemalavagu, M. Elaine Husni, Florian Rieder

**Affiliations:** 1 Department of Gastroenterology, Hepatology and Nutrition, Digestive Disease Institute, Cleveland Clinic Foundation, 9500 Euclid Ave / A30, Cleveland, OH 44195 USA; 2 Department of Musculoskeletal Radiology, Cleveland Clinic Foundation, Cleveland, OH USA; 3 Department of Rheumatologic and Immunologic Diseases, Cleveland Clinic Foundation, Cleveland, OH USA; 4 Cleveland Clinic Program for Global Translational Inflammatory Bowel Disease (GRID-IBD), Cleveland Clinic, Cleveland, OH USA; 5 Department of Inflammation and Immunity, Lerner Research Institute, Cleveland Clinic, Cleveland, OH USA

**Keywords:** Crohn’s disease, Ulcerative colitis, Inflammatory bowel disease, Inflammatory arthritis, Spondyloarthritis, Axial arthritis

## Abstract

**Purpose of Review:**

The goal of this review is to provide practical guidance for gastroenterologists regarding diagnosis and management of inflammatory bowel disease associated spondyloarthritis.

**Recent Findings:**

While there is limited data regarding optimal management approaches, we summarize available screening tools along with classification and consensus criteria that can be deployed in this setting and offer guidance regarding patient symptoms, physical exam findings, laboratory data, and imaging strategies that may facilitate diagnosis. In addition, we outline various therapeutic options available while highlighting deficits in the currently available literature.

**Summary:**

Inflammatory bowel disease associated spondyloarthritis is common but remains poorly understood. Ongoing research to establish a clear diagnostic pathway and reproducible treatment targets is essential to better identify and treat this patient population.

## Introduction

Inflammatory bowel diseases (IBD) are a group of chronic inflammatory disorders that classically involve the luminal gastrointestinal tract but also are associated with a number of extra-intestinal manifestations (EIMs) and other immune mediated inflammatory disorders (IMIDs). Inflammatory spondyloarthropathies (SpA) are among the most common of these manifestations and yet surprisingly there remains limited evidence-based guidance surrounding optimal approach to diagnosis and management [1–3]. This is of concern given impact on patient health-related quality of life as well as possible progression to disability without appropriate treatment [4–6]. In this review, we will provide a high-level overview of key research in this space with a focus on a practical approach to the diagnosis and management of IBD-associated axial (axSpA) and peripheral SpA (pSpA) for the gastroenterology provider.

IBD-pSpA is common, with prevalence rates as high as 40% in cohort studies [1]. As the name implies, IBD-pSpA involves the peripheral joints. The Cohort for Healing Arthritis, Skin, and Eye EIMs (CHASE-EIM), a multi-center prospective registry of patients with IBD and concomitant SpA led by our group, provides some insights into the pattern of peripheral joint involvement as well as association with luminal disease activity [7]. Preliminary data from 106 patients in this cohort found that 51 (49.5%) had fewer than five joints involved and 52 (50.5%) had *≥* 5 joints involved [7]. The most commonly involved joints were the hands (*n* = 72, 67.9%) followed by the knees (*n* = 56, 58.2%), with hand and knee involvement (*n* = 10, 9.4%) being the most commonly involved combination in the cohort [7]. Overall, IBD-pSpA was not associated with objective markers of luminal IBD disease activity, with no statistically significant association between pattern of joint distribution and markers of luminal disease activity including elevated fecal calprotectin (> 150 µg/g), active IBD on imaging, or active Crohn’s disease (CD) identified endoscopically [7]. Of note, the analysis did identify a statistically significant association between knee involvement and elevated C-reactive protein (CRP, odds ratio [OR] 4.3; 95% confidence interval [CI] 1.18–21.0; *p* = 0.04) and endoscopically active ulcerative colitis (UC) (OR, 5.9; 95% CI, 1.16– 37.0; *p* = 0.04) [7].

IBD-axSpA is less common than IBD-pSpA, with prevalence rates around 10% [8]. As the name implies, IBD-axSpA involves the spine and sacroiliac joints. IBD-axSpA runs independently of IBD disease activity. Early identification and treatment is essential due to the risk of progressive and debilitating complications if axSpA is left untreated [8].

Of note, both subtypes of SpA addressed in this review are distinguished from another common finding in IBD patients, arthralgia, by the presence of joint inflammation.

## Pathophysiology of IBD-SpA

While there remains uncertainty regarding the pathophysiology of EIMs in general and IBD-SpA in particular, there are two prevailing theories [9]. The first suggests that EIMs represent an extension of the luminal immune response [9]. Support for this theory includes data that shows the presence of T cells expressing gut-specific markers (for example, α4β7) in synovial fluid of patients with SpA, suggesting transit of gut-derived immune cells to joints [10]. The second posits the occurrence of EIMs as independent inflammatory events [9]. Support for this theory with respect to IBD-SpA includes specific patterns of gut dysbiosis seen in patients with SpA [11] as well as a number of shared genetic risk loci, including interleukin (IL)-23R for IBD and ankylosing spondylitis and nucleotide-binding oligomerization domain (NOD)2 for IBD and sacroiliitis [9, 12].

## Options for Screening for IBD-SpA in Gastroenterology Clinic

Given the prevalence, impact on patient health-related quality of life, and possibility of negative outcomes without treatment, early identification of patients with IBD-SpA or possible IBD-SpA who would benefit from referral to rheumatology is essential. To facilitate this, a number of screening tools have been proposed in the literature.

For the purposes of this review, we will focus on those specifically developed for patients with IBD. Options include the IBD Identification of Spondyloarthritis Questionnaire (IBIS-Q), the DETection of Arthritis in Inflammatory BoweL Diseases (DETAIL) questionnaire [13, 14], and a questionnaire developed by Quiero and colleagues [15]. The IBIS-Q includes 14 questions centered around pain, swelling, or stiffness in peripheral joints and the back as well as questions regarding difficulty with performance of everyday activities (such as tying shoes). The six item DETAIL questionnaire focuses on swollen or tender joints as well as persistent back pain that improves with exercise. The Queiro questionnaire asks three questions related to pSpA (the presence of joint pain, morning stiffness, and swelling) and three questions related to axSpA (back pain, morning stiffness, or pain that awakens the patient from sleep).

### Practical Approach to Screening in Clinic

Based on performance characteristics (> 80% post-test probability of SpA with three questionnaires answered affirmatively) as well as ease of administration, the DETAIL questionnaire may be the most practical choice, though confounding by fibromyalgia is of concern [14]. However, in a busy gastroenterology clinic, simply asking patients questions about the presence of inflammatory joint symptoms (peripheral joint pain associated with joint swelling and > 1 h of morning stiffness or back pain that worsens in the morning or with inactivity) can quickly cover many of the points raised in these screening questionnaires. Though not an evidenced-based recommendation, this is an approach routinely incorporated into our gastroenterology practice that in our anecdotal experience has facilitated identification of patients with possible IBD-SpA, especially as patients may not otherwise raise these symptoms during a visit with their gastroenterologist.

## Approach to Diagnosis and Work-Up of IBD-SpA in Gastroenterology Clinic

A number of classification and consensus criteria designed to assist in the diagnosis of SpA generally, and IBD-SpA in particular, have been published in both the gastroenterology and rheumatology literature [3]. Patient history, physical examination, laboratory data, and imaging may all also be incorporated into diagnostic approach [16]. Of note, a clear evidence-based path to diagnosis of IBD-pSpA in particular has not yet been established [16]. While there are still some limitations with respect to evidence-based approaches to the diagnosis of axSpA, there is nevertheless a robust body of literature regarding overall approach to axSpA diagnostics [2].

### pSpA Classification and Consensus Criteria

The Orchard classification schema is one that is familiar to many gastroenterologists [17]. In this schema, IBD-pSpA is split into two types [17]. Features of Type I include involvement of fewer than five joints, involvement of only large joints, asymmetrical joint distribution, association with IBD disease activity, and a duration of < 10 weeks [17]. Features of Type II include involvement of five or more joints, involvement of small joints, symmetrical joint involvement, lack of association with IBD disease activity, and a longer duration of months or years [17]. Given that this schema was based on a single center retrospective study that was not prospectively validated, is not routinely recommended within the rheumatology literature, is not consistent with CHASE findings, and is increasingly not recommended within the gastroenterology literature, we do not recommend use of this schema [3, 7, 16–18].

There are two consensus criteria developed by gastroenterologists available in lieu of the limitations of the Orchard criteria [16, 19]. The International Organization for the Study of IBD (IOIBD) developed criteria for use specifically in clinical trials [19]. These criteria advise utilization of rheumatologist expertise for diagnosis of IBD-pSpA [19]. If following these criteria, gastroenterologists should refer all patients with suspected IBD-pSpA to rheumatology. The CHASE group also developed consensus criteria for use specifically in clinical practice [16]. CHASE criteria require presence of swollen/tender joints on exam, a combination of morning stiffness and joint pain and swollen/tender joints on exam, and/or swollen/tender joints on exam with exclusion of other etiologies [16]. If following these criteria, gastroenterologists may make the diagnosis of IBD-pSpA but rheumatologic assessment is still considered optimal.

In addition, the European Crohn’s and Colitis Organization (ECCO) has published the only available guidelines regarding diagnosis of EIMs including IBD-SpA [18, 20]. ECCO guidelines endorse use of rheumatology criteria, specifically the ASAS criteria [18]. Originally developed for axSpA and later for pSpA, ASAS criteria require the presence of arthritis, enthesitis or dactylitis plus either one or more SpA features (uveitis, psoriasis, Crohn’s/colitis, preceding infection, HLA-B27, and sacroiliitis on imaging) or two more other SpA features (arthritis, enthesitis, dactylitis, inflammatory back pain, or family history of SpA) [21, 22]. Of note, arthritis is defined as inflammation of the joint space, enthesitis as inflammation at sites where tendons, ligaments, or joint capsules attach to bone, and dactylitis as swelling of the entire digit such that it has a sausage like appearance. Per these criteria, a patient with IBD as well as arthritis, enthesitis, or dactylitis could be diagnosed with IBD-pSpA. The overall sensitivity and specificity of the ASAS criteria are robust at 80% and 83%, respectively, however it is important to note that only a minority of patients included in the development of these criteria had IBD (3%) [21]. Of note, though there are also other rheumatology criteria available, given endorsement by ECCO as well as data that suggests superior performance of ASAS criteria we favor ASAS as the preferred criteria for IBD-pSpA from the rheumatology literature [3, 18].

### AxSpA Classification and Consensus Criteria

Though the Orchard schema is specific to only pSpA, the other screening criteria including IOIBD, CHASE, ECCO, and ASAS do include guidance for axSpA as well. IOIBD criteria define axSpA according to either rheumatologist expertise or inflammatory back/axial pain combined with typical magnetic resonance imaging (MRI) findings in a patient with IBD [16, 19]. Similarly, CHASE criteria for IBD-axSpA include patients who meet ASAS criteria as per rheumatologists and or patients with MRI, inflammatory back pain, and consistent MRI findings as per a rheumatologist [16].

ECCO guidelines again endorse use of the ASAS criteria [18]. For axSpA, ASAS criteria specify that patients with back pain > = 3 months and age at onset < 45 either have sacroiliitis on imaging and > = 1 SpA feature or HLA-B27 and > = 2 other SpA features [22]. Per ASAS, SpA features including IBP, arthritis, enthesitis (inflammation where a tendon attaches to bone), uveitis, dactylitis, psoriasis, IBD, good response to non-steroidal anti-inflammatory agents (NSAIDs), family history for SpA, HLA-B27, and elevated CRP [22].

For routine clinical practice, we recommend gastroenterologists familiarize themselves with the classification and consensus criteria listed above to enhance the ability to recognize possible concomitant SpA in an IBD patient. Key features common across criteria for diagnosis of IBD-pSpA include the presence of swollen or tender joints with morning stiffness and for axSpA include inflammatory back pain as well as consistent imaging (though it is important to note not all patients with axSpA will have radiographic findings). Input from rheumatology, especially for axSpA, remains essential.

### Practical Approach to Diagnosis of IBD-SpA

#### History

As already discussed, focused questions related to the presence of joint pain, joint swelling, morning stiffness, or IBP (all symptoms commonly reported in the IBD-SpA literature) may also help to enhance identification of patients with possible SpA [3]. For peripheral SpA, questions that we have found particularly helpful in our practice include assessment of the distribution and severity of the joint pain, relationship to luminal disease activity, duration of morning stiffness (with > 1 h of stiffness useful in differentiating inflammatory processes), response to NSAIDs, and response to IBD advanced therapies. In those patients who do endorse joint symptoms, focused additional questioning may help to eliminate alternative etiologies of joint pain (for example, the gastroenterologist may ask about widespread pain that may be more suggestive of fibromyalgia, recent infections that may be more suggestive of reactive arthritis or post viral syndromes, or worsening pain with movement which may be more suggestive of osteoarthritis). For axial SpA, assessment of whether the back pain awakes the patient from sleep (2nd half of the night), whether or not the pain improves with movement/exercise, a > 3 month duration of pain with insidious onset, and association with back stiffness > 1 h in the morning can be especially informative. In addition, inquiry regarding the presence of other EIMs, such as erythema nodosum, pyoderma gangrenosum, and uveitis/scleritis/episcleritis is beneficial as the presence of one EIM is associated with increased risk of other EIMs. Importantly, not all patients with IBD-axSpA will have symptoms and so the absence of symptoms alone cannot not rule out the presence of axSpA.

#### Physical Exam

The next diagnostic step for gastroenterologists involves a targeted musculoskeletal assessment to detect inflammatory arthritis and underscore its role as an EIM of IBD. A comparative inspection of the symptomatic joint and its contralateral counterpart should be performed to identify asymmetry, swelling, or erythema. This is followed by careful palpation to evaluate for effusion, tenderness, and increased warmth. Assessment of active and passive range of motion—and, when appropriate, grip strength—further characterizes joint involvement. Differential diagnostic considerations include monoarticular effusions with warmth and erythema suggestive of septic arthritis or gout; joint deformities characteristic of rheumatoid arthritis; and periarticular skin or nail changes indicative of psoriatic arthritis.

It is equally important to communicate clearly that articular symptoms may reflect the patient’s underlying IBD, thereby fostering patient insight and encouraging timely reporting of new manifestations. Because distinguishing between inflammatory and non-inflammatory joint disease can be challenging—and because axial involvement may coexist—a referral to rheumatology for a comprehensive peripheral and axial evaluation is advisable to ensure accurate diagnosis and optimal management.

#### Laboratory Work-Up

Though there is no laboratory data diagnostic for IBD-SpA, an elevated erythrocyte sedimentation rate (ESR) or C-reactive protein (CRP) may be suggestive but are nonspecific and confounding due to underlying luminal IBD disease activity is possible [16]. It is not unreasonable for the gastroenterologist to order a CRP as part of the diagnostic work-up. Depending on the history and physical examination findings as well as the comfort level of the gastroenterologist, laboratory testing to evaluate for alternative etiologies of joint disease such as rheumatoid factor may also be considered. Though HLA-B27 is included in ASAS criteria and may help in diagnosis of axSpA, this test may be best deferred to the rheumatologist given overall lower rates of positive HLA-B27 in IBD patients and the additional expertise brought by a rheumatologist in determining if this testing is truly necessary [3, 22, 23]. We do not routinely order extensive laboratory work-up for SpA in our clinical gastroenterology practice, but other bloodwork that may be helpful in identifying alternative etiologists includes rheumatoid factor and anti-cyclic citrullinated peptide antibodies (associated with rheumatoid arthritis).

#### Imaging

Finally, there is no imaging test alone that is currently considered diagnostic of SpA. While there are validated imaging findings in axSpA, the literature regarding imaging in IBD-pSpA is quite limited [16]. Based on this limited available literature, for IBD-pSpA, x-ray may be helpful for excluding alternative etiologies (like osteoarthritis) while joint MRI and joint ultrasound may be helpful in demonstrating features more specific to IBD-pSpA ​ [3, 14, 24–36]. When ordering imaging tests it is important to convey to radiologists the potential SpA diagnosis since imaging findings can be subtle. While ultrasound can be a well-tolerated and powerful tool in evaluating inflammatory arthropathies, before ordering, providers must understand the capabilities of the imaging department performing this testing. Many imaging groups may not have musculoskeletal ultrasound capability and, especially with ultrasound imaging, experience is very important. In general, ordering of imaging tests is best deferred to a rheumatologist who can select which is optimal for an individual patient. However, imaging tests may also be appropriately ordered by a gastroenterologist with experience in their interpretation and performance characteristics. When deciding which imaging test to order, the gastroenterologist should incorporate history, physical examination, and differential diagnosis to help prioritize which features they wish to assess. X-ray is most useful in evaluating for chronic degenerative changes and bony abnormalities, while ultrasound and MRI can more optimally assess for features suggestive of pSpA such synovitis or enthesitis. While higher cost, MRI likely provides the most detailed image of the joint and will allow for evaluation of bone marrow edema, and it is the optimal imaging test to assess specifically for axSpA. Gastroenterologists should remain vigilant regarding incidental note of the presence of sacroiliitis on imaging reports (such as computed tomography enterography) routinely obtained as part of standard of care for IBD patients.

#### Differential Diagnosis

Given the lack of a clear diagnostic path to IBD-pSpA, it is important to maintain a robust differential diagnosis (see Table 1). Throughout this review, we have highlighted features on history, exam, or laboratory data suggestive of alternative diagnoses. In general, for peripheral SpA the differential diagnosis may include septic arthritis, osteoarthritis, gout, rheumatoid arthritis, psoriatic arthritis, fibromyalgia, and medication side effects (for example, a lupus-like reaction to an anti-TNF agent). For axial spondyloarthritis, the differential commonly includes mechanical causes and degenerative disk disease. 

**Table 1:** Differential diagnosis for IBD-SpA

Peripheral SpA Differential Diagnosis:			
	Notable Clinical Features	Exam Findings	Other Relevant Data
Infectious Arthritis	• Acute • Monoarticular • Associated fever	• Warm, erythematous, tender joint • Limited range of motion	• Increased white blood cell count and inflammatory makers • Joint aspiration with evidence of infection (high white blood cell count, positive culture)
Osteoarthritis	• Chronic • Stiffness lasting <30 min • Worsens with movement	• Crepitus • Bony enlargement	• X-ray with joint space narrowing or osteophytes
Gout	• Acute • Monoarticular	• Exquisite joint tenderness • Classically involves 1st MTP joint	• Joint aspiration with monosodium urate crystals
Rheumatoid arthritis	• Chronic • Polyarticular • Often symmetric	• Classically involves MCPs and PIPs • Joint deformity present	• Elevated ESR/CRP • +RF, +CCP • Erosions on imaging
Psoriatic Arthritis	• Chronic • Polyarticular or Oligoarticular	• Classically involves DIP joints • May include nail pitting or psoriatic plaques	• Elevated ESR/CRP • Imaging may show periostitis and pencil in cup deformity
Fibromyalgia	• Chronic • Widespread pain	• Tenderness without objective joint swelling, erythema, or deformity	• Often associated with central sensitization but no objective abnormalities on work-up
Anti-TNF Induced Lupus	• Acute • Associated fever	• May have rash • May have serositis	• Elevated ESR/CRP • May have + ANA or anti-dsDNA
Axial SpA Differential Diagnosis			
Mechanical Back Pain	• Worsens with activity and improves with rest	• Paraspinal tenderness	• No inflammatory features
Degenerative Disk Disease	• Chronic pain • Worsens with movement • Possible radicular pain	• May have limited range of motion	• Imaging may show degenerative changes or disc space narrowing

SpA = spondyloarthritis, MTP = metatarsophalangeal joint, MCP = metacarpophalangeal joint, PIP = proximal interphalangeal joint, ESR = erythrocyte sedimentation rate, CRP = C reactive protein, RF = rheumatoid factor, CCP = cyclic citrullinated peptide, DIP = distal interphalangeal joints, ANA = antinuclear antibody, anti-dsDNA = ant-double stranded DNA

## Management

It is important to know that the optimal management of EIMs, especially IBD-pSpA, is not well studied. All recommendations that follow should thus be interpreted within that context. In general, guidance reported here is shaped by ECCO guidelines as well guidance included in a recent review article published by the authors [2, 18]. After initiation of treatment, patients should be closely followed by both gastroenterology and rheumatology to ensure improvement in joint and/or luminal disease activity with recommended follow-up intervals of at least every three months as per CHASE criteria [16].

### Practical Approach to Treatment of IBD-pSpA

For IBD-pSpA, we find that it is useful to begin with assessment of luminal disease activity. If there is objective evidence of active luminal disease as evidenced by an elevated fecal calprotectin, active disease on imaging including CT, MRI, or intestinal ultrasound (IUS), or active disease endoscopically, treating the underlying luminal inflammation and assessing for improved inflammation in both the lumen and the joints may be of benefit [2]. If there is no evidence of luminal disease, either use of an adjunctive therapy or a change to another luminal IBD therapy may be considered, depending on the severity of the joint symptoms and the luminal IBD disease history [2]. In general, this decision making must be tailored to each individual patient and is best conducted in collaboration with a rheumatologist.

Regarding available therapeutic options, there is mixed data with respect to 5-aminosalicylates. Sulfasalazine in particular may be considered in patients with inflammatory arthritis, but there is not strong data to support its use in enthesitis predominant disease [3, 18, 37, 38]. A multi-center randomized controlled trial evaluating sulfasalazine for axSpa and pSpA (not specifically related to IBD) noted that sulfasalazine was a safe and effective treatment for pSpA, while a more recent systematic review centered around treatment of enthesitis in psoriatic arthritis did not find benefit [3, 18, 37, 38]. While there is limited data to support methotrexate, this agent may also be utilized given some positive experience in the rheumatology literature as well as encouraging data from a small case series of 18 patients with UC published in Italy [2, 18, 39]. Of note, ECCO guidelines favor methotrexate over sulfasalazine, however especially given safety profile of sulfasalazine over methotrexate we use both agents in our clinical practice. While steroids are effective in symptom management, given side effect profile these are not recommended as maintenance therapy.

In regards to advanced therapies, it is important to note that no randomized controlled trials have been performed. The most robust data supports the use of anti-tumor necrosis factor (anti-TNF) therapies and JAK inhibitors (JAKi) [2, 18, 40–45]. Data supporting the role of anti-TNF agents includes resolution of arthritis in 75% of patients treated with adalimumab in an open label phase IIIb trial, a 73% response rate to anti-TNF therapy in the Swiss IBD cohort study, and statistically significant improvement in articular symptoms in a cohort of 24 patients with CD [41–44]. Data supporting use of JAKi includes post hoc analyses of two IBD maintenance trials demonstrating resolution (66.7% 30 mg, 38.5% 15 mg vs. 22.2% placebo, *P* = 0.010) and improvement (33.3% 10 mg twice daily, 16.7% 5 mg twice daily vs. 18.2% placebo, *P* value not provided) in IBD-pSpA at 52 weeks as well as a small open label prospective study (17 patients with pSpA and axSpA) and several case reports/series also demonstrating improvement in pSpA [40, 45–47]. Both ECCO guidelines and the authors favor use of anti-TNF therapies first-line given the more robust body of evidence as well as the fact that currently in the United States JAKi are only approved for disease refractory to anti-TNF therapies.

There is conflicting data regarding the use of ⍺4β7 with efficacy uncertain [2]. ECCO guidelines do not recommend use of vedolizumab for IBD-pSpA and warn about paradoxical arthritis activity [18]. However, a post HOC analysis from the GEMINI trial that showed reduced likelihood of new or worsening arthritis in patients with CD (hazard ratio 0.63; 95% confidence interval [CI], 0.44–0.89), data from a small prospective cohort study that did not find new features of SpA with vedolizumab treatment, and a multi-center real-world study found resolution of pSpA within 6 and 12 months (28.6% [*n* = 6 of 21] and 33.3% [*n* = 7 of 21]) [49–50]. Therefore, our recent review suggests vedolizumab may be considered in the management algorithm.

There is insufficient data surrounding IL-12/23 and IL-23 agents. Some studies with ustekinumab suggest lower rates of arthritis but one small study demonstrated no improvement in systemic joint symptoms [2, 18, 51, 52]. ECCO guidelines indicate that ustekinumab may be utilized. While risankizumab and guselkumab are approved for psoriatic arthritis, data for IBD-pSpA is insufficient to recommend their use [53, 54]. ECCO guidelines do not include these agents in their management algorithm, while we suggest that these agents may be considered with the understanding there is not yet enough data to formally recommend use. There is insufficient data for regarding sphingosine-1 receptor agents as well so these are also not recommended by ECCO guidelines or by the authors [2, 18].

### Practical Approach to Treatment of IBD-axSpA

For axSpA, the presence of luminal disease activity does not influence decision making. For axSpA, it is imperative to collaborate with rheumatology regarding treatment decisions [16]. While there are no randomized controlled trials dedicated to IBD-axSpA in particular, given approval of some anti-TNF agents and JAKi for treatment of both IBD and axSpA, these are the first line therapies [2, 18, 55–61]. All other advanced therapies are not indicated for treatment of both IBD and axSpA and are not recommended by either ECCO guidelines or the authors. However, in some cases one therapy for IBD and an alternative therapy for axSpA may be required.

### NSAIDs

Emerging data has suggested that use of NSAIDs may not be as strongly associated with flares of luminal IBD as was previously thought [18]. Given this, short courses of selective cyclooxygenase-2 inhibitors such as celecoxib may be considered in selected patient populations with significant joint symptoms as a bridge to a long-term therapeutic choice.

## Future Directions

Given the limited data in this space, our group is leveraging the CHASE cohort to investigate risk factors for development of IBD-pSpA, efficacy of various available treatment options, relationship to luminal IBD disease activity, and impact on patient quality of life and work productivity. We are also in the process of a developing a patient reported outcome to facilitate assessment of treatment response in clinical trials. Additionally, a global RAND/UCLA panel composed of experts in rheumatology, gastroenterology, and radiology led by our group is currently underway to establish definitions, diagnosis, and monitoring strategies for clinical trials. Finally, we are exploring the efficacy of various imaging modalities with a focus on joint ultrasound given the growing accessibility of ultrasound in gastroenterology clinics as IUS becomes part of standard of care for assessment of IBD patients.

## Conclusions

IBD-SpA is commonly encountered in clinical practice though there is limited guidance available in the published literature. There are various validated screening tools available to improve identification of possible IBD-SpA patients. Similarly, there are a number of classification and consensus criteria available for use in gastroenterology clinic. Of these, ECCO guidelines along with the CHASE and ASAS criteria may be easiest to deploy for the busy practicing clinical gastroenterologist. Patient history, physical exam findings, laboratory data, and imaging findings can be used in combination to support diagnosis, with input from rheumatologist crucial in successfully and accurately establishing a diagnosis of IBD-SpA. For IBD-pSpA, there are a number of treatment options available with selection guided by underlying IBD disease activity and ideally input from rheumatology. More research is needed across therapies to better understand positioning, particularly with respect to some of the novel advanced therapies for IBD. For IBD-axSpA, anti-TNF agents or JAKi should be selected first-line, and all treatment decisions should be made in multi-disciplinary fashion with rheumatology. IBD-SpA remains an unmet need and ongoing research in this space is essential to improving IBD-SpA outcomes.

## Key References

- Gordon H, Burisch J, Ellul P, et al. ECCO Guidelines on Extraintestinal Manifestations in Inflammatory Bowel Disease. J Crohns Colitis 2024;18:1–37.
-
  - The European Crohn’s and Colitis Organization has published the only available guidelines on extra-intestinal manifestations of IBD, including IBD-associated spondyloarthritis. This recent guideline update is thus an important reference for all questions regarding extra-intestinal manifestations in IBD.
- Falloon K, Forney M, Husni ME, et al. Diagnosis and Management of Inflammatory Bowel Disease-Associated Spondyloarthritis. American Journal of Gastroenterology 2024.
-
  - This review article published by our group provides a comprehensive overview regarding diagnosis and management of IBD-associated SpA.
- Falloon K, Dossaji Z, Mude P, et al. Diagnosis of Inflammatory Bowel Diseases-Associated Peripheral Spondyloarthritis: A Systematic Review. Inflammatory Bowel Diseases Journal 2024.
-
  - This systematic review published by our group summarizes the data surrounding all available diagnostic parameters for IBD-associated pSpA.
- Rudwaleit M, Heijde D van der, Landewe R, et al. The Assessment of SpondyloArthritis international Society classification criteria for peripheral spondyloarthritis and for spondyloarthritis in general. Ann Rheum Dis 2011;70:25–31.
- Rudwaleit M, Heijde D van der, Landewe R, et al. The development of Assessment of SpondyloArthritis international Society classification criteria for axial spondyloarthritis (part II): validation and final selection. Ann Rheum Dis 2009;68:777–783.
-
  - While these are both older publications, they are essential as they outline the validated rheumatology criteria for diagnosis of axial and peripheral inflammatory spondyloarthropathy. Of note, these criteria are endorsed by ECCO guidelines.

## Data Availability

No datasets were generated or analysed during the current study.
